# The effect of lipidomes on the risk of endometrioid endometrial cancer: a Mendelian randomization study

**DOI:** 10.3389/fonc.2024.1436955

**Published:** 2024-10-18

**Authors:** Yaochen Lou, Feng Jiang, Jun Guan

**Affiliations:** ^1^ Department of Gynecology, Obstetrics and Gynecology Hospital of Fudan University, Shanghai, China; ^2^ Department of Neonatology, Obstetrics and Gynecology Hospital of Fudan University, Shanghai, China

**Keywords:** Mendelian randomization, endometrioid endometrial cancer, lipidome, GWAS, Finnish cohort

## Abstract

**Objective:**

This study aimed to explore the potential effects between various human plasma lipidomes and endometrioid endometrial cancer (EEC) by using Mendelian randomization (MR) methods.

**Methods:**

This study designated a total of 179 human plasma lipidomes from the genome-wide association study (GWAS) database as the exposure variable. An EEC-related dataset from the GWAS (GCST006465) served as the outcome variable. MR analyses used the inverse variance-weighted method (IVW), MR-Egger, weighted median, simple mode, and weighted mode methods for regression calculations, accounting for possible biases induced by linkage disequilibrium and weak instrument variables. Any lipidomes failing to pass heterogeneity and horizontal pleiotropy tests were deemed to lack significant causal impact on the outcome.

**Results:**

The results of IVW analysis disclosed that a variety of human plasma lipidomes (n = 15) exhibited a significant causal effect on EEC (p < 0.05). A subset of these lipidomes (n = 13) passed heterogeneity and horizontal pleiotropy tests, which demonstrated consistent and viable causal effects (p < 0.05) including glycerophospholipids, glycerolipids, and sterols. Specifically, phosphatidylcholine (odds ratio [OR]: 1.065-1.129, p < 0.05) exhibited a significant positive causal effect on the occurrence of EEC. Conversely, sterol ester (OR = 0.936, p = 0.007), diacylglycerol (OR = 0.914, p = 0.036), phosphatidylcholine (OR: 0.903-0.927, p < 0.05), phosphatidylethanolamine (OR = 0.907, p = 0.046) and triacylglycerol (OR: 0.880-0.924, p < 0.05) showed a notable negative causal association with EEC, suggesting their inhibitory effects on the EEC occurrence.

**Conclusions:**

The study revealed that human plasma lipidomes have complex impacts on EEC through Mendelian randomization. This indicated that the diversity of structural changes in lipidomes could show different effects on subtypes and then affect EEC occurrence. Although these lipids had the potential to be promising biomarkers, they needed to be further clinically validated nevertheless.

## 1 Introduction

The global incidence of endometrial cancer (EC) is rising significantly (1–3), and accounts for 4.5% of all female cancers, with 417,367 new cases and 97,370 EC-related deaths reported (1, 2, 4). Within it, the endometrioid endometrial cancer (EEC) is the main histotype, comprising approximately 80% of EC (3). Given EC’s severity and prevalence, it is essential to explore the underlying etiology to reduce its occurrence, which is also helpful to develop effective and personalized treatments for susceptible groups.

Lipid metabolism is one of the most commonly dysregulated metabolic pathways in EC (5), which has significant implications for oncogenesis and tumor progression (6, 7). Recent advances in lipidomics have greatly expanded our understanding of the complexities and range of circulating lipids. Currently, human plasma lipid species could be divided into four categories as glycerophospholipids, glycerolipids, sphingolipids, and sterols (8). Shifts in lipids can impact critical cellular processes such as proliferation, apoptosis, migration, and invasion, all of which are essential in the development and progression of cancers (9–12). Also, the alterations in the lipid composition of cell membranes may modify the activity of associated receptors and signaling pathways, thereby influencing cancer cell growth (11, 13). Moreover, lipids link to energy metabolism and tumorigenesis-related inflammation (10, 14), both of which are considered to play an important role in cancer pathogenesis (15). Supportingly, some studies have indicated that endometrial cancer cells may potentially possess unique lipid signatures, and improve the risk prediction for EC (16–18). It is noteworthy that liposomes have biocompatible and biodegradable characteristics, which can render them various platforms for drug delivery (19). Liposomes are spherical vesicles made of one or more concentric phospholipid bilayers encasing an aqueous core and play the role of lipid-based nanocarriers (19, 20). They bolster the therapeutic efficacy by stabilizing compounds, facilitating cross-membrane transport, and enhancing drugs’ pharmacokinetics. Additionally, liposomes provide precision in targeting drug delivery to specific bodily sites and weaken systemic toxicity (21). Maybe it is beneficial for novel therapeutic drug delivery. However, at present our knowledge of liposomes and EC is deficient, and further investigation is necessary to clarify these mechanisms.

Mendelian randomization (MR) provides a method to infer causality from the associations between exposures and outcomes, leveraging genetic variants as instrumental variables (IVs) (22). Observational studies could indicate an association between specific lipidomes and increased EC risk, however, the potential for residual confounding should be acknowledged (22). At present, the causal link between lipidomes and EC susceptibility remains an open question.

In this study, genetic data from Finnish cohorts were utilized to identify genetic determinants affecting the human plasma lipidomes, which were analyzed by using two-sample MR methods. The research aimed to discern genetic associations between various lipidomes and the development of EEC.

## 2 Materials and methods

### 2.1 Data sources and genetic variant selection

The exposure dataset sourced from the genome-wide association study (GWAS) Catalog (https://www.ebi.ac.uk/gwas/, GCST90277238-GCST90277416) included a total of 179 human plasma lipidomes in 7,174 Finnish individuals from GeneRISK cohort, which consisted of 4,579 females and 2,595 males (8). This GeneRISK cohort comprised 7,342 participants (4,691 females and 2,651 males), recruited from southern Finland during 2015-2017, aged 45-66 years with an average age of 56 (23). According to its baseline health survey, 11.1% of the individuals received lipid-lowering treatment and 22.0% received antihypertensive treatment (23). The 179 lipidomes were classified into 13 lipid classes encompassing 4 major categories, which included glycerolipids, glycerophospholipids, sphingolipids, and sterols (8). The dataset associated with EEC was identified by ebi-a-GCST006465 in the GWAS database (http://gwas.mrcieu.ac.uk/datasets/). A genome-wide significance threshold of p < 5×10^-8^ was used to identify single nucleotide polymorphisms (SNPs) strongly associated with 179 human plasma lipidomes and EEC. Nevertheless, due to a limited amount of selected SNPs for some lipidomes when utilizing human plasma lipidomes as the exposure, the threshold was adjusted to 1×10^-5^ to identify the top independent SNPs, which has been commonly used in prior MR studies (24, 25). To be in line with the Mendelian second law for independence among SNPs, the parameters for linkage disequilibrium (LD) were set as r^2^ < 0.001 and kb > 10,000. SNPs were selected to ensure they were independent, thus reducing potential confounding effects from LD. If the LD r^2^ value equaled or exceeded 0.001, one of the SNPs was excluded from further analysis. Additionally, a genetic distance of 10,000 kb was set to represent the region’s length. Within this 10,000 kb region, SNPs with an r^2^ value above 0.001 were removed to eliminate any remaining LD. These stringent selection criteria were applied to meet the assumptions of MR and ensure a robust and valid evaluation of the lipidome’s impact on EEC. SNPs chosen on this basis were regarded as Instrumental Variables (IVs) for subsequent analyses. Lipidomes lacking adequate SNPs for MR analysis were dropped from the study.

To ensure the accuracy of results, in this study we adopted the F-statistic as a measure of the association degree between IVs and the exposure. To minimize concerns over weak instrument bias, an F-statistic of ≤ 10 was set for weak IVs.

### 2.2 Statistical analysis for MR

The MR analysis in this study primarily was based on the “TwoSampleMR” package in R software (version 4.3.2). Other R packages were used for further analysis and graphical presentation of results, including “ggplot2”, “ComplexHeatmap”, “circlize”, “dendextend”, “dendsort”, “gridBase”, “tidyverse”, and “ggforestplot”.

The inverse-variance weighted (IVW) method was a meta-analysis that combines variant-specific Wald ratios for each mutation with an assumption that each mutation satisfies IV criteria (22). It integrated the estimated effects across different SNPs, providing a precise estimate of the effect of exposure on outcome (22). Including a large number of genetic variants could increase statistical power, however, there was a risk that they may not qualify as valid IVs (26). And importantly, the outcomes of IVW served as our principal reference.

To mitigate potential biases introduced by individual models, the weighted median and MR-Egger methods were employed as supplementary and reference approaches to the IVW model.

Pleiotropy meant a potential association between genetic variants and multiple phenotypes (27). To assess pleiotropy, MR-Egger and weighted median approaches were used as pleiotropy-robust methods. Through a weighted linear regression model, MR-Egger regression could correct for the bias due to directional pleiotropy considering the effects of multiple genetic variants across all instruments (26). The slope coefficient represented a causal effect estimation (26). The intercept represented the average pleiotropic effect estimated across genetic variants (26). To further comprehensively evaluate the pleiotropic effects, the Mendelian Randomization Pleiotropy RESidual Sum and Outlier (MR-PRESSO) test also was used which could identify and correct for potential outliers (28).

The weighted median approach consistently estimated causal effects even with 50% of the information derived from valid IVs (29). It showed higher accuracy compared to the MR-Egger approach (29). To estimate the true causal effect, we also used the weighted mode approach as an additional MR method. It assigned weights to variants, with the largest weights assigned to valid IVs among all variant subgroups (30). Although its power for detecting a causal effect in a two-sample setting was lower than that of IVW and weighted median approaches, it outperformed MR-Egger regression (30), with similar precision to that of the IVW and weighted median approaches (30).

If the causal effect directions are consistent across these models, it suggests a relatively stable causal effect between the exposure and the outcome. Cochran’s *Q*-statistics and *I* ^2^ statistics were also used to examine heterogeneity in this study (31–33).

In all test methods mentioned, a significance level of p < 0.05 was considered statistically significant.

## 3 Results

### 3.1 The data and detailed information

**Figure 1:**
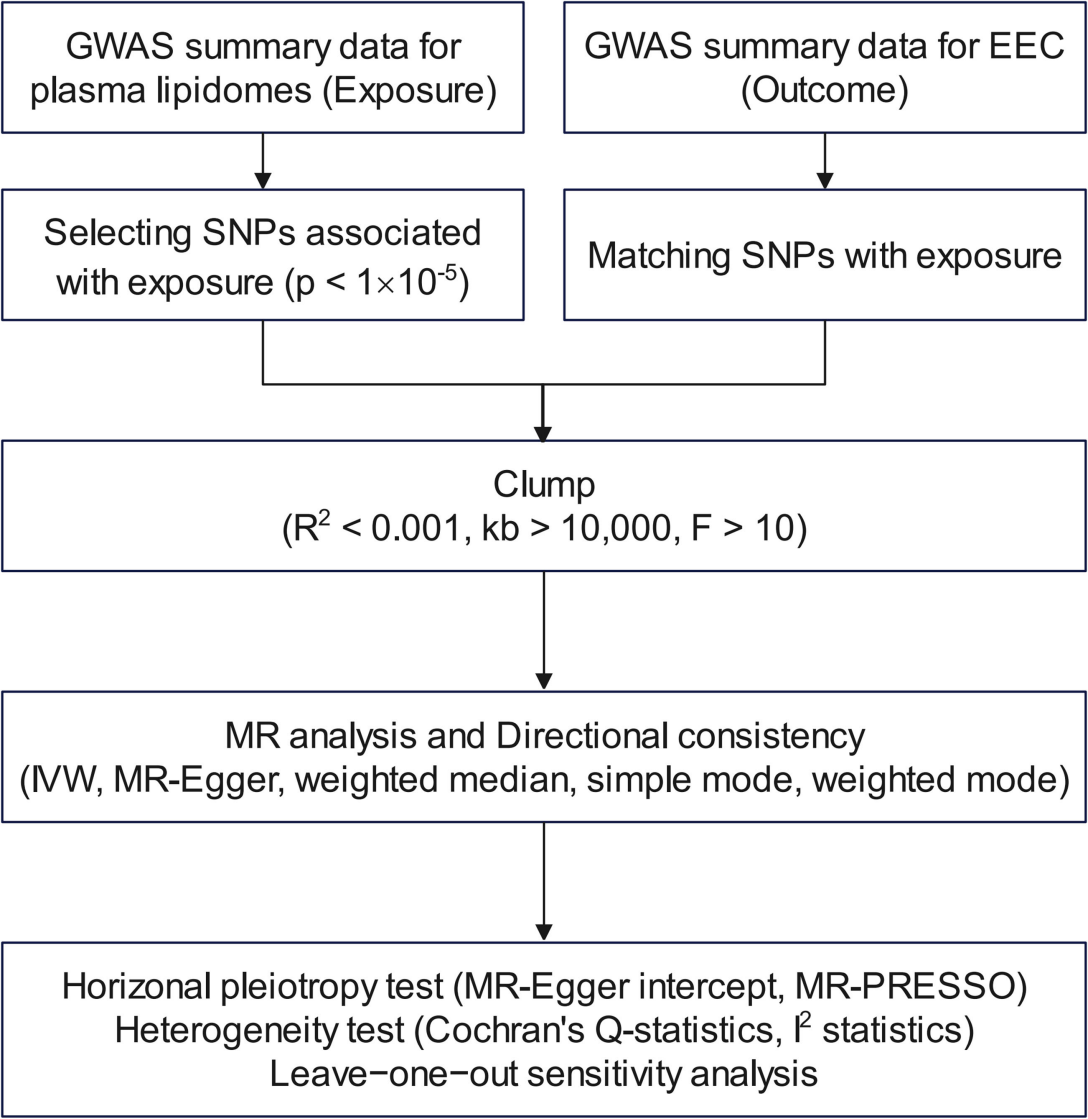
Flow chart for filtering lipidomes. GWAS, genome-wide association study; EEC, endometrioid endometrial cancer; SNP, single-nucleotide polymorphism; MR, Mendelian randomization; IVW, inverse-variance weighted; MR-PRESSO, Mendelian Randomization Pleiotropy RESidual Sum and Outlier.

The GWAS dataset used in this study is primarily based on the genetic locus study conducted by Linda Ottensmann et al. in 2023, which investigated the impact of genetic variations on 179 human plasma lipidomes (8). All the datasets and the EEC-related dataset were based on sequencing performed on individuals of European ancestry. Our analysis incorporated 54,884 individuals of European descent, comprised of 8,758 cases and 46,126 controls, all with endometrioid endometrial cancer histology. The lipidome selection was shown as a flow chart visually in **Figure 1**.

### 3.2 The IVs selection for MR analysis

The GWAS dataset for all lipidomes was screened for SNPs based on the threshold of p < 1×10^-5^. The parameters for LD were set as r^2^ < 0.001 and kb > 10,000. If a lipidome had an insufficient number of SNPs for MR analysis, it was excluded from the study. The SNPs selected based on the criterion of F statistic > 10 were presented in detail in **Supplementary Table S1**. The F statistics for all SNPs ranged from 19.53 to 1819.85.

### 3.3 The results of MR analysis

**Figure 2:**
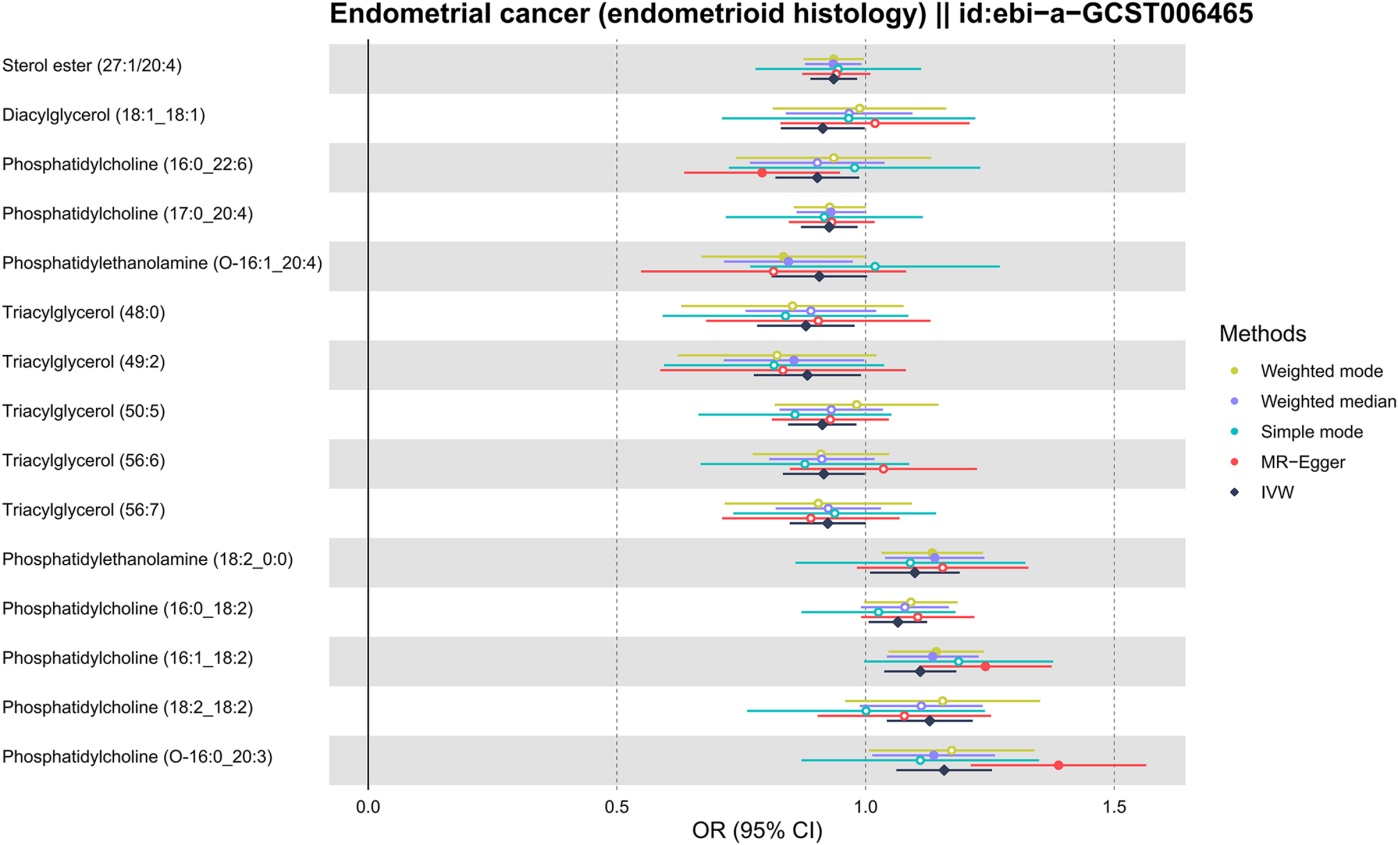
Forest plot for MR results of 15 lipidomes. MR, Mendelian randomization; IVW, inverse-variance weighted; OR, odds ratio; CI, confidence interval.

**Figure 3:**
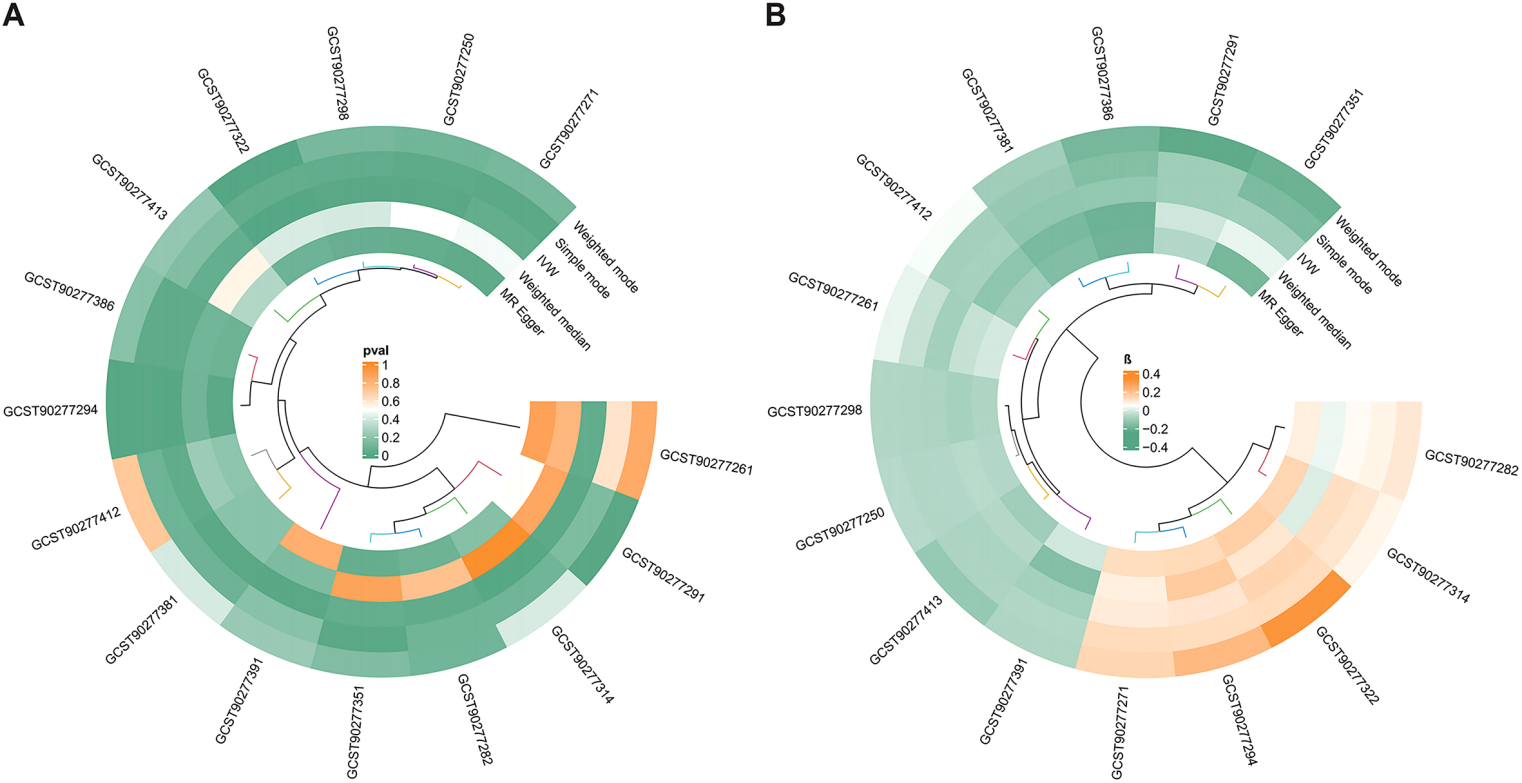
The circular heatmap shows the p-values and β-values of algorithms corresponding to 15 lipidomes. **(A)** Heatmap of p-values corresponding to 15 lipidomes. **(B)** Heatmap of β-values corresponding to 15 lipidomes. The clustering algorithm used in the circular heatmap is an unsupervised clustering method, which simply represents the similarity among the data points. MR, Mendelian randomization; IVW, inverse-variance weighted.

As shown in **Figure 2**, the MR analysis revealed a potential effect between human plasma lipidomes and EEC, including glycerophospholipids, glycerolipids, and sterols. With a p-value of IVW < 0.05, five were found with potential positive effects while ten had potential negative effects. **Table 1** disclosed the details of the odds ratio (OR) and 95% confidence interval (CI) between 15 lipidomes and EEC. A lower incidence of EEC was associated with sterol ester (27:1/20:4), diacylglycerol (18:1_18:1), phosphatidylcholine (16:0_22:6), phosphatidylcholine (17:0_20:4), phosphatidylethanolamine (O-16:1_20:4), triacylglycerol (48:0), triacylglycerol (49:2), triacylglycerol (50:5), triacylglycerol (56:6), and triacylglycerol (56:7). Conversely, phosphatidylethanolamine (18:2_0:0), phosphatidylcholine (16:0_18:2), phosphatidylcholine (16:1_18:2), phosphatidylcholine (18:2_18:2), phosphatidylcholine (O-16:0_20:3) exhibited a positive association with EEC. **Figure 3** visually presented a circular heatmap illustrating the p-values and β-values of IVW, MR-Egger and weighted median methods corresponding to the aforementioned lipidomes (**Figures 3A, B**). Among them, 15 lipidomes showed consistent causal effects with the IVW direction based on the weighted median and MR-Egger.

**Table 1:** The MR results of 15 lipidomes.

GCST ID	lipidomes	MR methods	SNP number	OR (95% CI)	p-value	SE
GCST90277250	Sterol ester (27:1/20:4)	MR-Egger	27	0.941 (0.878-1.008)	0.095	0.035
		Weighted median	27	0.935 (0.883-0.990)	0.021	0.029
		IVW	27	0.936 (0.893-0.982)	0.007	0.024
		Simple mode	27	0.945 (0.800-1.115)	0.505	0.085
		Weighted mode	27	0.936 (0.882-0.995)	0.042	0.031
GCST90277261	Diacylglycerol (18:1_18:1)	MR-Egger	23	1.019 (0.842-1.233)	0.851	0.097
		Weighted median	23	0.967 (0.851-1.098)	0.602	0.065
		IVW	23	0.914 (0.840-0.994)	0.036	0.043
		Simple mode	23	0.966 (0.748-1.248)	0.795	0.130
		Weighted mode	23	0.988 (0.829-1.177)	0.893	0.089
GCST90277291	Phosphatidylcholine (16:0_22:6)	MR-Egger	23	0.792 (0.677-0.927)	0.008	0.080
		Weighted median	23	0.903 (0.788-1.035)	0.142	0.069
		IVW	23	0.903 (0.831-0.981)	0.016	0.043
		Simple mode	23	0.978 (0.759-1.261)	0.868	0.129
		Weighted mode	23	0.936 (0.770-1.138)	0.513	0.100
GCST90277298	Phosphatidylcholine (17:0_20:4)	MR-Egger	27	0.932 (0.856-1.015)	0.119	0.044
		Weighted median	27	0.930 (0.869-0.995)	0.036	0.035
		IVW	27	0.927 (0.876-0.981)	0.008	0.029
		Simple mode	27	0.917 (0.752-1.118)	0.398	0.101
		Weighted mode	27	0.928 (0.864-0.998)	0.054	0.037
GCST90277351	Phosphatidylethanolamine (O-16:1_20:4)	MR-Egger	21	0.815 (0.624-1.063)	0.148	0.136
		Weighted median	21	0.845 (0.742-0.963)	0.011	0.066
		IVW	21	0.907 (0.824-0.998)	0.046	0.049
		Simple mode	21	1.019 (0.793-1.309)	0.886	0.128
		Weighted mode	21	0.835 (0.708-0.986)	0.046	0.084
GCST90277381	Triacylglycerol (48:0)	MR-Egger	16	0.905 (0.723-1.134)	0.401	0.115
		Weighted median	16	0.890 (0.780-1.015)	0.082	0.067
		IVW	16	0.880 (0.799-0.970)	0.010	0.050
		Simple mode	16	0.839 (0.655-1.074)	0.184	0.126
		Weighted mode	16	0.853 (0.683-1.066)	0.183	0.114
GCST90277386	Triacylglycerol (49:2)	MR-Egger	14	0.834 (0.652-1.067)	0.175	0.126
		Weighted median	14	0.856 (0.743-0.986)	0.031	0.072
		IVW	14	0.883 (0.793-0.984)	0.024	0.055
		Simple mode	14	0.816 (0.654-1.018)	0.095	0.113
		Weighted mode	14	0.822 (0.674-1.004)	0.077	0.102
GCST90277391	Triacylglycerol (50:5)	MR-Egger	23	0.929 (0.827-1.044)	0.231	0.060
		Weighted median	23	0.931 (0.840-1.032)	0.174	0.053
		IVW	23	0.913 (0.853-0.977)	0.008	0.035
		Simple mode	23	0.858 (0.706-1.042)	0.136	0.099
		Weighted mode	23	0.982 (0.832-1.158)	0.830	0.084
GCST90277412	Triacylglycerol (56:6)	MR-Egger	26	1.036 (0.859-1.250)	0.716	0.096
		Weighted median	26	0.912 (0.821-1.013)	0.086	0.054
		IVW	26	0.916 (0.845-0.994)	0.035	0.042
		Simple mode	26	0.878 (0.712-1.084)	0.237	0.107
		Weighted mode	26	0.910 (0.793-1.045)	0.195	0.070
GCST90277413	Triacylglycerol (56:7)	MR-Egger	32	0.890 (0.745-1.064)	0.210	0.091
		Weighted median	32	0.925 (0.832-1.029)	0.153	0.054
		IVW	32	0.924 (0.856-0.998)	0.045	0.039
		Simple mode	32	0.938 (0.765-1.150)	0.543	0.104
		Weighted mode	32	0.905 (0.750-1.092)	0.305	0.096
GCST90277271	Phosphatidylethanolamine (18:2_0:0)	MR-Egger	26	1.155 (0.972-1.371)	0.115	0.088
		Weighted median	26	1.139 (1.031-1.257)	0.010	0.051
		IVW	26	1.099 (1.003-1.203)	0.043	0.046
		Simple mode	26	1.090 (0.866-1.373)	0.469	0.118
		Weighted mode	26	1.134 (1.024-1.257)	0.024	0.052
GCST90277282	Phosphatidylcholine (16:0_18:2)	MR-Egger	34	1.105 (0.987-1.238)	0.094	0.058
		Weighted median	34	1.079 (0.989-1.178)	0.088	0.045
		IVW	34	1.065 (1.005-1.129)	0.033	0.030
		Simple mode	34	1.026 (0.879-1.198)	0.749	0.079
		Weighted mode	34	1.091 (0.994-1.198)	0.076	0.048
GCST90277294	Phosphatidylcholine (16:1_18:2)	MR-Egger	24	1.241 (1.085-1.418)	0.005	0.068
		Weighted median	24	1.135 (1.035-1.245)	0.007	0.047
		IVW	24	1.110 (1.033-1.193)	0.004	0.037
		Simple mode	24	1.187 (0.981-1.437)	0.092	0.097
		Weighted mode	24	1.142 (1.038-1.256)	0.012	0.049
GCST90277314	Phosphatidylcholine (18:2_18:2)	MR-Egger	23	1.078 (0.906-1.283)	0.405	0.089
		Weighted median	23	1.112 (0.983-1.258)	0.092	0.063
		IVW	23	1.129 (1.036-1.230)	0.006	0.044
		Simple mode	23	1.001 (0.788-1.272)	0.994	0.122
		Weighted mode	23	1.155 (0.948-1.406)	0.166	0.100
GCST90277322	Phosphatidylcholine (O-16:0_20:3)	MR-Egger	16	1.388 (1.164-1.655)	0.003	0.090
		Weighted median	16	1.137 (1.004-1.287)	0.043	0.063
		IVW	16	1.158 (1.053-1.273)	0.003	0.049
		Simple mode	16	1.110 (0.873-1.410)	0.408	0.122
		Weighted mode	16	1.173 (0.994-1.385)	0.079	0.085

MR, Mendelian randomization; IVW, inverse-variance weighted; OR, odds ratio; CI, confidence interval; SNP, single-nucleotide polymorphism; SE, standard error.

### 3.4 The test results of heterogeneity and horizontal pleiotropy

Furthermore, by using Cochran’s *Q* test and *I* ^2^ statistics, MR-Egger and MR-PRESSO methods, a number of 13 lipidomes successfully passed tests for heterogeneity and horizontal pleiotropy, and exhibited the correct causal effect direction. Phosphatidylcholine (16:0_22:6) [p = 0.6302921 (MR Egger); p = 0.4599443 (IVW)], phosphatidylcholine (17:0_20:4) [p = 0.6323824 (MR Egger); p = 0.6837995 (IVW)], phosphatidylethanolamine (O-16:1_20:4) [p = 0.2323808 (MR Egger); p = 0.2429853 (IVW)], triacylglycerol (48:0), [p = 0.7215898 (MR Egger); p = 0.7792433 (IVW)], triacylglycerol (49:2), [p = 0.6593042 (MR Egger); p = 0.7135652 (IVW)], triacylglycerol (50:5), [p = 0.3876029 (MR Egger); p = 0.4387764 (IVW)], triacylglycerol (56:6), [p = 0.1297905 (MR Egger); p = 0.0964322 (IVW)], triacylglycerol (56:7), [p = 0.1932159 (MR Egger); p = 0.2206511 (IVW)], dacylglycerol (18:1_18:1) [p = 0.6068697 (MR Egger); p = 0.5702879 (IVW)], and sterol ester (27:1/20:4) [p = 0.5728314 (MR Egger); p = 0.6262988 (IVW)] had negative directions. Besides, phosphatidylcholine (16:0_18:2) [p = 0.6862283 (MR Egger); p = 0.7050082 (IVW)], phosphatidylcholine (16:1_18:2) [p = 0.4202402 (MR Egger); p = 0.2840796 (IVW)], and phosphatidylcholine (18:2_18:2) [p = 0.2144235 (MR Egger); p = 0.2419252 (IVW)] had forward directions. **Table 2** displays the details of 13 lipidomes that passed the tests along with their respective p-values in heterogeneity and horizontal pleiotropy analyses.

**Table 2:** Details of 13 lipidomes that passed the test.

GCST ID	Lipidomes	p-value of heterogeneity (MR-Egger)	p-value of heterogeneity (IVW)	p-value of horizontal pleiotropy	Direction
GCST90277282	Phosphatidylcholine (16:0_18:2)	0.6862283	0.7050082	0.4660940	Forward
GCST90277294	Phosphatidylcholine (16:1_18:2)	0.4202402	0.2840796	0.0718408	Forward
GCST90277314	Phosphatidylcholine (18:2_18:2)	0.2144235	0.2419252	0.5574212	Forward
GCST90277250	Sterol ester (27:1/20:4)	0.5728314	0.6262988	0.8503631	Negative
GCST90277261	Diacylglycerol (18:1_18:1)	0.6068697	0.5702879	0.2278977	Negative
GCST90277291	Phosphatidylcholine (16:0_22:6)	0.6302921	0.4599443	0.0679216	Negative
GCST90277298	Phosphatidylcholine (17:0_20:4)	0.6323824	0.6837995	0.8613317	Negative
GCST90277351	Phosphatidylethanolamine (O-16:1_20:4)	0.2323808	0.2429853	0.4074765	Negative
GCST90277381	Triacylglycerol (48:0)	0.7215898	0.7792433	0.7880618	Negative
GCST90277386	Triacylglycerol (49:2)	0.6593042	0.7135652	0.6237871	Negative
GCST90277391	Triacylglycerol (50:5)	0.3876029	0.4387764	0.7115147	Negative
GCST90277412	Triacylglycerol (56:6)	0.1297905	0.0964322	0.1688780	Negative
GCST90277413	Triacylglycerol (56:7)	0.1932159	0.2206511	0.6486125	Negative

MR, Mendelian randomization; IVW, inverse-variance weighted.

### 3.5 Scatter plots of MR analysis

**Figure 4:**
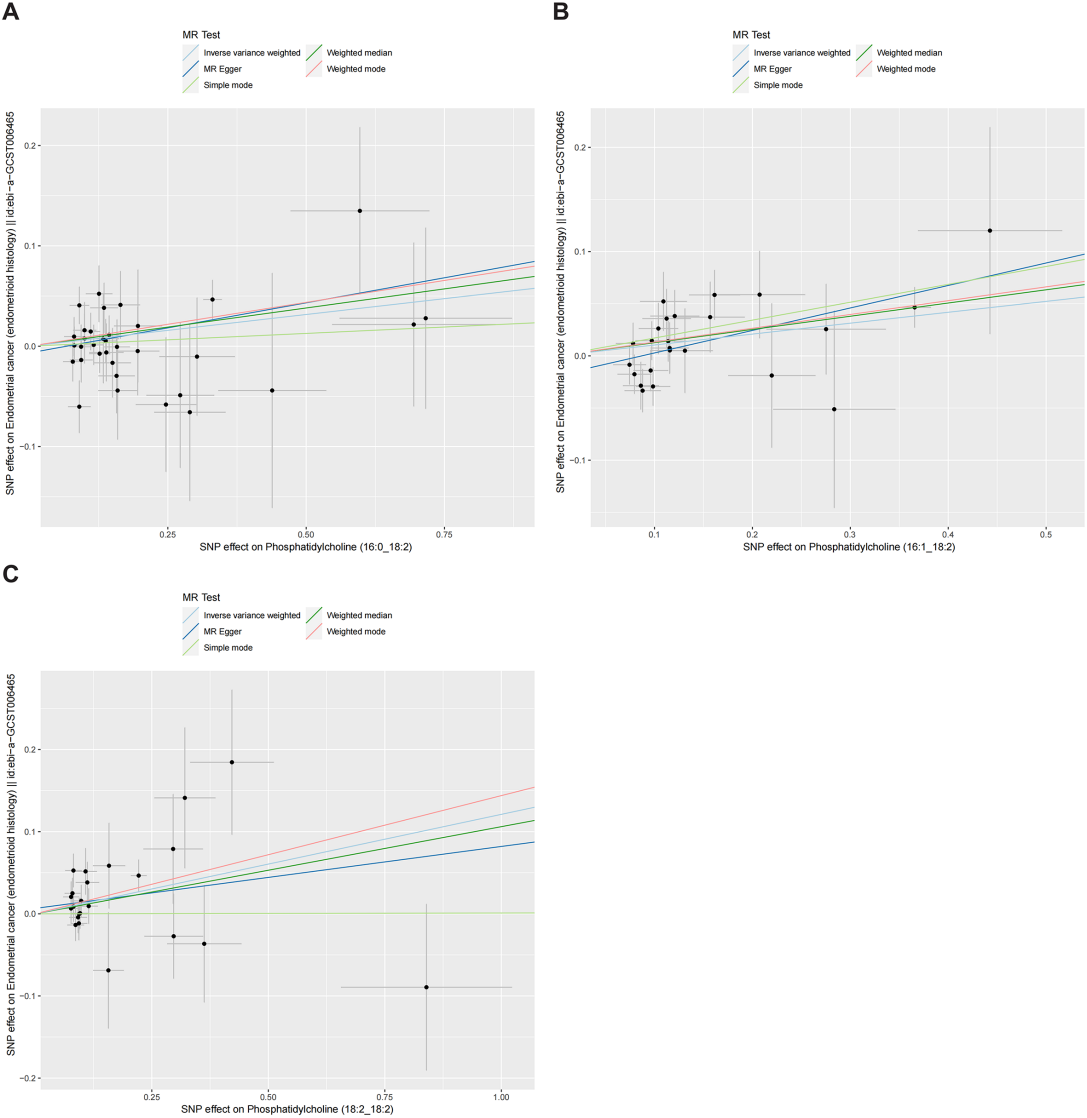
Scatter plots of three lipidomes with forward direction. The slopes of each line represented the causal association for each method. The light blue line represented the IVW estimate, the dark green line represented the weighted median estimate, the dark blue line represented the MR-Egger estimate, the light green line represented the simple mode estimate, and the pink line represented the weighted mode estimate. IVW, inverse-variance weighted; MR, Mendelian randomization; SNP, single-nucleotide polymorphism. **(A)** Phosphatidylcholine (16:0_18:2). **(B)** Phosphatidylcholine (16:1_18:2). **(C)** Phosphatidylcholine (18:2_18:2).

**Figure 5:**
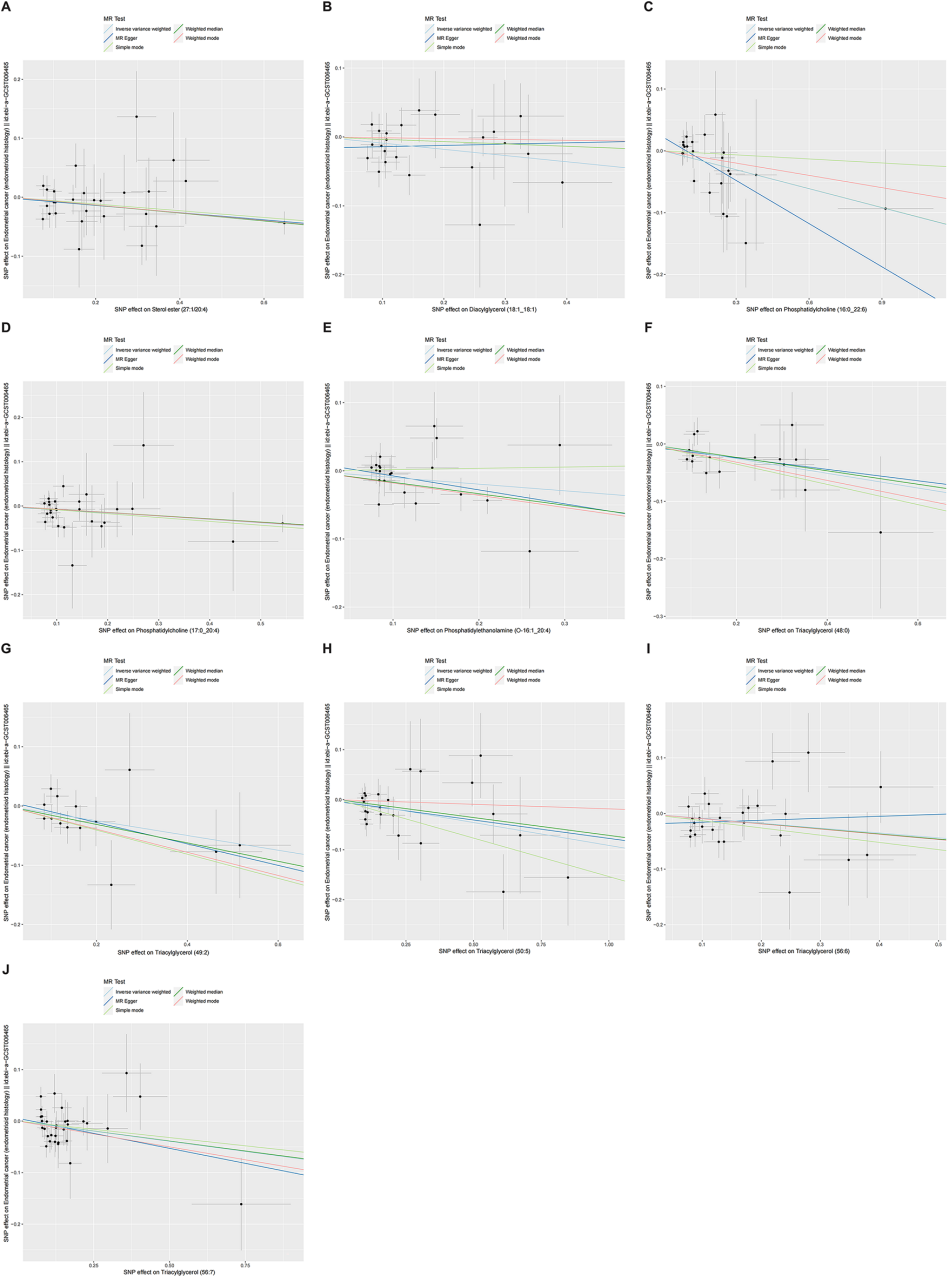
Scatter plots of ten lipidomes with negative direction. The slopes of each line represented the causal association for each method. The light blue line represented the IVW estimate, the dark green line represented the weighted median estimate, the dark blue line represented the MR-Egger estimate, the light green line represented the simple mode estimate, and the pink line represented the weighted mode estimate. IVW, inverse-variance weighted; MR, Mendelian randomization; SNP, single-nucleotide polymorphism. **(A)** Sterol ester (27:1/20:4). **(B)** Diacylglycerol (18:1_18:1). **(C)** Phosphatidylcholine (16:0_22:6). **(D)** Phosphatidylcholine (17:0_20:4). **(E)** Phosphatidylethanolamine (O-16:1_20:4). **(F)** Triacylglycerol (48:0). **(G)** Triacylglycerol (49:2). **(H)** Triacylglycerol (50:5). **(I)** Triacylglycerol (56:6). **(J)** Triacylglycerol (56:7).

**Figures 4**, **5** presented the regression results of the SNPs corresponding to the aforementioned 13 lipidomes using the IVW, weighted median, and MR-Egger algorithms. **Figure 4** indicated that phosphatidylcholine (16:0_22:6), phosphatidylcholine (17:0_20:4), phosphatidylethanolamine (O-16:1_20:4), triacylglycerol (48:0), triacylglycerol (49:2), triacylglycerol (50:5), triacylglycerol (56:6), triacylglycerol (56:7), diacylglycerol (18:1_18:1) and sterol ester (27:1/20:4) showed significant negative causal associations with EEC. Conversely, phosphatidylcholine (16:0_18:2), phosphatidylcholine (16:1_18:2), and phosphatidylcholine (18:2_18:2) exhibited significant positive causal effects on EEC.

## 4 Discussion

In this study, we used MR methods to explore the complex causal associations between 179 human plasma lipidomes and the risk of EEC. Our findings implied that EEC disease was linked with lipidomes and discovered significant effects of glycerophospholipids, glycerolipids, and sterols on EEC risk. Several triacylglycerol (TG) variants (49:2, 50:5, 56:6, and 56:7) and diacylglycerol (DG) within the glycerolipids group, sterol ester (SE) within the sphingolipids group, phosphatidylethanolamine (PE) and phosphatidylcholines (PCs) within the glycerophospholipids group exhibited as protective roles for EEC. While the effects of PCs varied depending on their structural compositions, a few variants of phosphatidylcholine (16:0_18:2, 16:1_18:2, and 18:2_18:2) showed positive roles for EEC.

This study was the first to investigate the causal effects of various lipidomes with different structures on EEC occurrence in a relatively homogenous population, and it illustrated the divergent roles of lipidome subtypes. The findings revealed the complexity of lipidomes, which could be negative or positive other than traditional perspectives. As promising biomarkers for EEC, they played a potential role in disease managements and might impact patients’ prognosis and survival. Moreover, it benefited mechanistic studies, pharmaceutical developments, and the advancement of precision medicine, all of which hold significant clinical importance and value.

Traditionally, DGs and TGs were considered to increase the risk of numerous diseases. However, in our study, DG and several forms of TGs with different structures might be protective factors for EEC. Similarly, some studies investigating lipid metabolism and tumors also indicated an inverse association between TGs and oncogenesis. Eiji Hishinuma et al. quantified 628 metabolites in plasma samples from 142 EC patients by using ultra-high-performance liquid chromatography coupled with tandem mass spectrometry (16). They found reduced levels of TGs (TG 20:5_34:0, 20:5_36:2, 22:6_32:1, and 22:6_34:2), which included polyunsaturated fatty acids (PUFA), in these patients (16). These findings are consistent with our results, indicating that TGs containing unsaturated fatty acids play a crucial role in EC progression. Hang Zheng et al. demonstrated that the omega-3 PUFA could markedly inhibit cell proliferation, induce cell cycle arrest, and trigger apoptosis in cellular and animal models with EC (34). Besides, previous research showed that omega-3 PUFA supplementation may mitigate the harmful effects of obesity-driven cancers (35). These findings indicated the metabolic effects of omega-3 PUFAs in EC tumors. However, as with many complex diseases, the common variants identified via GWAS account for only a small portion of the heritability of tumors, and rare variants throughout the genome might also significantly influence disease progression (36). Consequently, an integrated approach that combines fundamental research with clinical investigations is essential to clarify the causal links more comprehensively (36).

We found PE also had an observed protective effect on EEC. PE species were integral to cellular survival, signaling pathways, and apoptotic processes. Abnormal metabolism of PEs has been observed in various cancers (37, 38), with a significant decrease in hepatic, pulmonary, gastric, and colorectal cancers (39). Also, Agnieszka Skorupa et al. discovered that PE levels are reduced in ECs of all grades compared to normal endometrial tissue (40). The biosynthesis of PE was identified as a key pathway that was dysregulated in EC when compared to normal tissue as well as among tumors of different grades (40). The abundance of PE levels was proved to positively regulate longevity in both yeast and mammalian cells, which was related to the induction of autophagy (41, 42). Through precursor ethanolamine supplementation in mammalian cells, the enhancement of endogenous PEs led to an observable increase in autophagic flux measured by LC3 lipidation (41). It partly interpreted the negative correlation between PE and EEC in our study and was conducive to finding potential therapeutic targets in the future.

SEs were the unsaturated sterols esterified with long-chain fatty acids and served as a storage form for these fatty acids, buffering against excess sterols that might otherwise interfere with essential cellular functions (43, 44). In humans, cholesterol was the most abundant sterol lipid. As levels of SEs increased, levels of free sterols could be reduced. By maintaining the proper level of esterified cholesterol, membrane stability might be supported, potentially reducing the risk of EEC tumorigenesis. Besides, in the synthesis of SEs, enzymes could mediate the esterification of cholesterol on the surface of high-density lipoproteins (HDL), which were absorbed from peripheral tissue (45). HDL was an acknowledged good lipid protein with antioxidant and anti-inflammatory properties and played a role in preventing oxidative modification of low-density lipoproteins (LDL) (46). Oxidized LDL was implicated in inducing mutagenesis, promoting cellular proliferation, and initiating metastasis (47). This suggested that insufficient SE could lead to insufficient HDL and elevate the risk of oxidative and inflammatory exposure, contributing to the development and progression of EEC potentially. Additionally, Avery Sengupta et al. found that the inclusion of sterol esters in the diet of hypercholesterolemic rats led to a reduction in plasma cholesterol and an increase in antioxidant enzyme levels in cell membranes (48). It was well known that reactive oxygen species (ROS) generated by cellular alterations could promote the secretion of inflammatory factors, while excessive inflammation could amplify oxidative stress, causing cell and tissue damage and potentially leading to cancer (49). This oxidative stress could activate the PI3K/AKT/mTOR signaling pathway, which was involved in EC proliferation and progression (50). The interplay between oxidative stress and chronic inflammation established a vicious cycle that supported EC development (50). These findings suggested that sterol esters might help reduce the risk of EEC, presenting an intriguing and worthy question for future research.

The effects of PCs were inconsistent, with different structural forms playing completely antagonistic roles. Both negative and positive associations revealed a complex relationship between PCs and EEC. Similar findings were also revealed in a European prospective investigation (51). PCs were the predominant glycerophospholipids in eukaryotic membranes and were linked to tumor cell proliferation and signal transduction (52). Multiple studies have identified PCs as potential diagnostic biomarkers. Knific et al. analyzed 126 plasma samples and identified three reduced PC levels in EC patients (53). It observed that plasma PC was lower in patients with EC than in healthy volunteers (53). This reduction might reflect oncogenic alterations in lipid transporter and PC metabolism enzyme activities. Several studies on endometrial cancer tissue reported on the enzymes degrading PCs. Metabolic enzymes, phospholipases A1 (PLA1) and A2 (PLA2), which converted PC to lysophosphatidylcholine (LPC) and then metabolized to lysophosphatidic acid (LPA) by lysophospholipase D (LPD) (54). PLA2 and LPD were overexpressed in cancerous tissues compared to normal endometrium, leading to an increased formation of LPC and LPA (55). Similar significant decreases in plasma PC levels have been reported in patients with ovarian and cervical cancers (38, 56, 57). On the other hand, PC remodeling might influence the level of HDL in EC patients (58). With higher levels of PC variants, HDL had a significant decrease, and HDL was negatively correlated with transcription factor EB and estrogen-related receptor α (58). As high expressions of both were associated with cancer progression and poor prognosis of EC (58), it might partly explain the positive relationship between PCs and EEC based on enhanced membrane fluidity. Certainly, the precise mechanisms at play remained to be further explored. Deeper investigations were necessary to assess the potential clinical significance of these findings.

Several observational studies have suggested an association between disease severity and lipid profiles. For instance, Chen et al. found that patients with type 2 diabetes had higher levels of total cholesterol (TC), TG, and low-density lipoprotein cholesterol (LDL-C) in the observation group compared to the control group, while high-density lipoprotein cholesterol (HDL-C) levels were lower (59). Additionally, TC, TG, and LDL-C levels were lower in the mild group compared to the moderate and severe groups, whereas HDL-C levels were higher in the mild group (59). This research indicated that the severity of type 2 diabetes correlates with more profound lipid metabolism disorders. Similarly, Nie et al. discovered that in Alzheimer’s disease (AD) patients, lipid metabolite changes accompanied the progression from mild to moderate to severe stages (60). Specifically, the relative content of TG (18:0_16:0_18:0)+NH4 and TG (18:0_16:0_16:0)+NH4 increased with AD severity, while PE (20:0_20:4)-H and LPC (16:1e)-CH3 decreased (60). Regarding EC, only a few studies have been conducted. Lorentzen et al. found that fatty acids, glycerophospholipids, and sphingolipids were enriched in EC through metabolomic analysis, suggesting a role for lipids in disease progression and severity, warranting further research (61). Therefore, analyzing the lipid profile concerning disease severity might provide deeper insights into the metabolic disruptions in patients, thereby enhancing the robustness and applicability of study findings, and offering more tailored interventions and management strategies based on disease severity. However, the GWAS database used in our study did not provide specific clinical pathological characteristics of the patients.

Lipidomes play interconnected roles in various biological processes, acting not only as structural constituents of cellular membranes but also as signaling molecules and energy sources. In the context of endometrial cancer, dysregulated lipid metabolism significantly influences cancer initiation and progression through mechanisms that are interdependent and collectively contribute to tumor growth, survival, and metastasis. For example, cholesterol, the most abundant sterol lipid in humans, is intricately linked to synthesizing steroid hormones such as estrogens, progestogens, androgens, and corticosteroids (62, 63). Elevated cholesterol levels can enhance the production of estrogens, impacting the progression of various hormone-responsive tumors, including EC, breast cancer, colorectal cancer, and others (64). Cholesterol is converted into pregnenolone, the precursor of all steroid hormones, and subsequently into estrogens like estradiol, which promotes the proliferation of endometrial cells (65, 66). The interplay between cholesterol-derived estrogens and lipid signaling pathways facilitates cancer growth by activating estrogen receptor signaling in cancer cells (67). Additionally, oxysterols can act as estrogen mimetics by binding to estrogen receptors, further connecting cholesterol metabolism with estrogen-driven carcinogenesis (67). These multifaceted interactions underscore the important role of lipid metabolism in EC and highlight potential avenues for therapeutic intervention.

The relationship between lipidomes and EEC involves complex biochemical pathways. As lipid metabolism is closely linked to cancer development and progression, one of the key pathways is the interplay between Lipids and estrogen synthesis. Adipose tissue can be the primary site for aromatase enzyme activity, which converts androgens (such as androstenedione) into estrogens, directly promoting endometrial proliferation and the transcription of pro-proliferative genes, thereby supporting the growth of estrogen-sensitive endometrial cells (68, 69). Estrogens bind to estrogen receptors in endometrial cells, directly regulating the transcription of multiple pro-proliferative genes, including insulin-like growth factor 1 (IGF1) and IGF1R, and activating the MAPK and AKT signaling pathways that stimulate endometrial cell proliferation (68, 70). Estrogen-mediated gene products ultimately regulate autophagy, proliferation, apoptosis, survival, differentiation, and vasodilation (70). Moreover, Lipids influences chronic inflammation associated with visceral fat and can promote hyperinsulinemia, increased IGF1, and hyperglycemia (71). Insulin and IGFs can promote cell proliferation and survival by activating pathways such as the PI3K/AKT/mTOR pathway (72, 73). This activation leads to increased cell growth and survival while inhibiting apoptosis, contributing to cancer development (72, 73). Additionally, high insulin levels can also reduce sex hormone-binding globulin, further contributing to the elevated levels of free estrogens (74). These interconnected pathways underscore the role of lipid metabolism and its influence on hormone regulation and inflammatory pathways in the pathogenesis of EC.

This research has several strengths. It is the first study to apply the MR approach to comprehensively explore the link between plasma lipidomes and EEC. By using MR analysis, it minimizes bias from unobserved confounding to the greatest extent possible, effectively enhancing the validity of our conclusions. The considerable sample size amplifies the study’s statistical robustness, facilitating a more accurate determination of causal relationships. Furthermore, by conducting investigations within a relatively homogeneous population, our findings can be extrapolated to the population more precisely. Despite these strengths, our study also has some limitations. Firstly, it focuses on the endometrioid subtype of EC and exclusively encompasses participants of European descent, which may lessen the applicability of our results to other ethnic groups and EC subtypes. Secondly, because lacking of clinical data in GWAS datasets and no access requests are currently accepted in THL Biobank, we did not analyze the effect of clinical factors such as age, stage, grade, molecular classification and drug treatments history on the lipid profile (75). Thirdly, we applied a less stringent p-value threshold of 1×10^-5^ (rather than the standard 5×10^-8^) during the SNP screening of lipid profiles to ensure a sufficient number of SNPs referenced to previous MR studies (24, 25). Besides, due to the difficulty in obtaining high-quality, high-confidence SNPs, we did not perform multivariable Mendelian randomization (MVMR) analysis that might allow the disentanglement of the direct effects of each lipid trait on an outcome while accounting for pleiotropy within lipid pathways. Notably, MR analysis cannot entirely rule out all confounding effects on other biological pathways related to EEC phenotypes, and our results does not clarify the mechanisms or molecular pathways by which metabolites may affect the development of EC. Thus, future studies should aim to refine these limitations through basic and clinical studies to unravel the complex interaction between plasma lipidomes and EC.

## 5 Conclusion

In conclusion, applying a two-sample MR study, we found the complex effects of human plasma lipidomes on EEC. The diversity in structural alterations of lipidomes may have different impacts on subtypes, subsequently influencing the occurrence of EEC. As promising biomarkers, these lipidomes are worthy of further clinical validation.

## Data Availability

The original contributions presented in the study are included in the article/**Supplementary Material**. Further inquiries can be directed to the corresponding authors.

## References

[1] Sung H Ferlay J Siegel RL Laversanne M Soerjomataram I Jemal A. Global cancer statistics 2020: GLOBOCAN estimates of incidence and mortality worldwide for 36 cancers in 185 countries. CA Cancer J Clin. (2021) 71:209–49. doi: 10.3322/caac.21660 33538338

[2] Zhang S Gong TT Liu FH Jiang YT Sun H Ma XX. Global, regional, and national burden of endometrial cancer, 1990-2017: results from the global burden of disease study, 2017. Front Oncol. (2019) 9:1440. doi: 10.3389/fonc.2019.01440 31921687 PMC6930915

[3] Lu KH Broaddus RR. Endometrial cancer. N Engl J Med. (2020) 383:2053–64. doi: 10.1056/NEJMra1514010 33207095

[4] Bray F Ferlay J Soerjomataram I Siegel RL Torre LA Jemal A. Global cancer statistics 2018: GLOBOCAN estimates of incidence and mortality worldwide for 36 cancers in 185 countries. CA Cancer J Clin. (2018) 68:394–424. doi: 10.3322/caac.21492 30207593

[5] Li W Xu Y Zeng X Tan J Wang Y Wu H. Etiological relationship between lipid metabolism and endometrial carcinoma. Lipids Health Dis. (2023) 22:116. doi: 10.1186/s12944-023-01868-2 37537560 PMC10401764

[6] Menendez JA Lupu R. Fatty acid synthase and the lipogenic phenotype in cancer pathogenesis. Nat Rev Cancer. (2007) 7:763–77. doi: 10.1038/nrc2222 17882277

[7] Cairns RA Harris IS Mak TW. Regulation of cancer cell metabolism. Nat Rev Cancer. (2011) 11:85–95. doi: 10.1038/nrc2981 21258394

[8] Ottensmann L Tabassum R Ruotsalainen SE Gerl MJ Klose C Widen E. Genome-wide association analysis of plasma lipidome identifies 495 genetic associations. Nat Commun. (2023) 14:6934. doi: 10.1038/s41467-023-42532-8 37907536 PMC10618167

[9] Zhang F Du G. Dysregulated lipid metabolism in cancer. World J Biol Chem. (2012) 3:167–74. doi: 10.4331/wjbc.v3.i8.167 PMC3430731 22937213

[10] Martin-Perez M Urdiroz-Urricelqui U Bigas C Benitah SA. The role of lipids in cancer progression and metastasis. Cell Metab. (2022) 34:1675–99. doi: 10.1016/j.cmet.2022.09.023 36261043

[11] Hisano Y Hla T. Bioactive lysolipids in cancer and angiogenesis. Pharmacol Ther. (2019) 193:91–8. doi: 10.1016/j.pharmthera.2018.07.006 PMC6309747 30048709

[12] Luo X Zhao X Cheng C Li N Liu Y Cao Y. The implications of signaling lipids in cancer metastasis. Exp Mol Med. (2018) 50:1–10. doi: 10.1038/s12276-018-0150-x PMC6154999 30242145

[13] Park JB Lee CS Jang JH Ghim J Kim YJ You S. Phospholipase signalling networks in cancer. Nat Rev Cancer. (2012) 12:782–92. doi: 10.1038/nrc3379 23076158

[14] Koundouros N Poulogiannis G. Reprogramming of fatty acid metabolism in cancer. Br J Cancer. (2020) 122:4–22. doi: 10.1038/s41416-019-0650-z 31819192 PMC6964678

[15] Hanahan D Weinberg RA. Hallmarks of cancer: the next generation. Cell. (2011) 144:646–74. doi: 10.1016/j.cell.2011.02.013 21376230

[16] Hishinuma E Shimada M Matsukawa N Shima Y Li B Motoike IN. Identification of predictive biomarkers for endometrial cancer diagnosis and treatment response monitoring using plasma metabolome profiling. Cancer Metab. (2023) 11:16. doi: 10.1186/s40170-023-00317-z 37821929 PMC10568780

[17] Njoku K Campbell AE Geary B MacKintosh ML Derbyshire AE Kitson SJ. Metabolomic biomarkers for the detection of obesity-driven endometrial cancer. Cancers (Basel). (2021) 13:e1–e23. doi: 10.3390/cancers13040718 PMC7916512 33578729

[18] Njoku K Sutton CJ Whetton AD Crosbie EJ. Metabolomic biomarkers for detection, prognosis and identifying recurrence in endometrial cancer. Metabolites. (2020) 10:e1–e28. doi: 10.3390/metabo10080314 PMC7463916 32751940

[19] Shah S Dhawan V Holm R Nagarsenker MS Perrie Y. Liposomes: Advancements and innovation in the manufacturing process. Adv Drug Delivery Rev. (2020) 154-155:102–22. doi: 10.1016/j.addr.2020.07.002 32650041

[20] Liu G Hou S Tong P Li J. Liposomes: preparation, characteristics, and application strategies in analytical chemistry. Crit Rev Anal Chem. (2022) 52:392–412. doi: 10.1080/10408347.2020.1805293 32799645

[21] Guimaraes D Cavaco-Paulo A Nogueira E. Design of liposomes as drug delivery system for therapeutic applications. Int J Pharm. (2021) 601:120571. doi: 10.1016/j.ijpharm.2021.120571 33812967

[22] Sanderson E Glymour MM Holmes MV Kang H Morrison J Munafo MR. Mendelian randomization. Nat Rev Methods Primers. (2022) 2:6. doi: 10.1038/s43586-021-00092-5 37325194 PMC7614635

[23] Widen E Junna N Ruotsalainen S Surakka I Mars N Ripatti P. How communicating polygenic and clinical risk for atherosclerotic cardiovascular disease impacts health behavior: an observational follow-up study. Circ Genom Precis Med. (2022) 15:e003459. doi: 10.1161/CIRCGEN.121.003459 35130028

[24] Wei Z Xiong Q Huang D Wu Z Chen Z. Causal relationship between blood metabolites and risk of five infections: a Mendelian randomization study. BMC Infect Dis. (2023) 23:663. doi: 10.1186/s12879-023-08662-6 37805474 PMC10559484

[25] Sanna S van Zuydam NR Mahajan A Kurilshikov A Vich Vila A Vosa U. Causal relationships among the gut microbiome, short-chain fatty acids and metabolic diseases. Nat Genet. (2019) 51:600–5. doi: 10.1038/s41588-019-0350-x PMC6441384 30778224

[26] Bowden J Davey Smith G Burgess S. Mendelian randomization with invalid instruments: effect estimation and bias detection through Egger regression. Int J Epidemiol. (2015) 44:512–25. doi: 10.1093/ije/dyv080 PMC4469799 26050253

[27] Stearns FW. One hundred years of pleiotropy: a retrospective. Genetics. (2010) 186:767–73. doi: 10.1534/genetics.110.122549 PMC2975297 21062962

[28] Verbanck M Chen CY Neale B Do R. Detection of widespread horizontal pleiotropy in causal relationships inferred from Mendelian randomization between complex traits and diseases. Nat Genet. (2018) 50:693–8. doi: 10.1038/s41588-018-0099-7 PMC6083837 29686387

[29] Bowden J Davey Smith G Haycock PC Burgess S. Consistent estimation in mendelian randomization with some invalid instruments using a weighted median estimator. Genet Epidemiol. (2016) 40:304–14. doi: 10.1002/gepi.21965 PMC4849733 27061298

[30] Hartwig FP Davey Smith G Bowden J. Robust inference in summary data Mendelian randomization via the zero modal pleiotropy assumption. Int J Epidemiol. (2017) 46:1985–98. doi: 10.1093/ije/dyx102 PMC5837715 29040600

[31] Higgins JP Thompson SG. Quantifying heterogeneity in a meta-analysis. Stat Med. (2002) 21:1539–58. doi: 10.1002/sim.1186 12111919

[32] Bowden J Del Greco MF Minelli C Davey Smith G Sheehan NA Thompson JR. Assessing the suitability of summary data for two-sample Mendelian randomization analyses using MR-Egger regression: the role of the I2 statistic. Int J Epidemiol. (2016) 45:1961–74. doi: 10.1093/ije/dyw220 PMC5446088 27616674

[33] Egger M Smith GD Phillips AN. Meta-analysis: principles and procedures. BMJ. (1997) 315:1533–7. doi: 10.1136/bmj.315.7121.1533 PMC2127925 9432252

[34] Zheng H Tang H Liu M He M Lai P Dong H. Inhibition of endometrial cancer by n-3 polyunsaturated fatty acids in preclinical models. Cancer Prev Res (Phila). (2014) 7:824–34. doi: 10.1158/1940-6207.capr-13-0378-t 24866178

[35] Khatib SA Rossi EL Bowers LW Hursting SD. Reducing the burden of obesity-associated cancers with anti-inflammatory long-chain omega-3 polyunsaturated fatty acids. Prostaglandins Other Lipid Mediat. (2016) 125:100–7. doi: 10.1016/j.prostaglandins.2016.07.011 PMC5064866 27448716

[36] Cao Y Ai M Liu C. The impact of lipidome on breast cancer: a Mendelian randomization study. Lipids Health Dis. (2024) 23:109. doi: 10.1186/s12944-024-02103-2 38622701 PMC11017498

[37] Cheng M Bhujwalla ZM Glunde K. Targeting phospholipid metabolism in cancer. Front Oncol. (2016) 6:266. doi: 10.3389/fonc.2016.00266 28083512 PMC5187387

[38] Cheng F Wen Z Feng X Wang X Chen Y. A serum lipidomic strategy revealed potential lipid biomarkers for early-stage cervical cancer. Life Sci. (2020) 260:118489. doi: 10.1016/j.lfs.2020.118489 32976882

[39] Lee GB Lee JC Moon MH. Plasma lipid profile comparison of five different cancers by nanoflow ultrahigh performance liquid chromatography-tandem mass spectrometry. Anal Chim Acta. (2019) 1063:117–26. doi: 10.1016/j.aca.2019.02.021 30967175

[40] Skorupa A Ponski M Ciszek M Cichon B Klimek M Witek A. Grading of endometrial cancer using (1)H HR-MAS NMR-based metabolomics. Sci Rep. (2021) 11:18160. doi: 10.1038/s41598-021-97505-y 34518615 PMC8438077

[41] Rockenfeller P Koska M Pietrocola F Minois N Knittelfelder O Sica V. Phosphatidylethanolamine positively regulates autophagy and longevity. Cell Death Differ. (2015) 22:499–508. doi: 10.1038/cdd.2014.219 25571976 PMC4326582

[42] Ichimura Y Kirisako T Takao T Satomi Y Shimonishi Y Ishihara N. A ubiquitin-like system mediates protein lipidation. Nature. (2000) 408:488–92. doi: 10.1038/35044114 11100732

[43] Korber M Klein I Daum G. Steryl ester synthesis, storage and hydrolysis: A contribution to sterol homeostasis. Biochim Biophys Acta (BBA) - Mol Cell Biol Lipids. (2017) 1862:1534–45. doi: 10.1016/j.bbalip.2017.09.002 28888831

[44] Sturley SL. Molecular aspects of intracellular sterol esterification: the acyl coenzyme A: cholesterol acyltransferase reaction. Curr Opin Lipidol. (1997) 8:167–73. doi: 10.1097/00041433-199706000-00007 9211065

[45] O K Hill JS Wang X Pritchard PH. Recombinant lecithin:cholesterol acyltransferase containing a Thr123–>Ile mutation esterifies cholesterol in low density lipoprotein but not in high density lipoprotein. J Lipid Res. (1993) 34:81–8. doi: 10.1016/s0022-2275(20)41321-5 8445345

[46] Barter PJ Nicholls S Rye KA Anantharamaiah GM Navab M Fogelman AM. Antiinflammatory properties of HDL. Circ Res. (2004) 95:764–72. doi: 10.1161/01.res.0000146094.59640.13 15486323

[47] Bitorina AV Oligschlaeger Y Shiri-Sverdlov R Theys J. Low profile high value target: The role of OxLDL in cancer. Biochim Biophys Acta Mol Cell Biol Lipids. (2019) 1864:158518. doi: 10.1016/j.bbalip.2019.158518 31479734

[48] Sengupta A Ghosh M. Effect of sterol esters on lipid composition and antioxidant status of erythrocyte membrane of hypercholesterolemic rats. J Oleo Science. (2014) 63:439–47. doi: 10.5650/jos.ess13211 24770475

[49] Vallee A Lecarpentier Y. Crosstalk between peroxisome proliferator-activated receptor gamma and the canonical WNT/beta-catenin pathway in chronic inflammation and oxidative stress during carcinogenesis. Front Immunol. (2018) 9:745. doi: 10.3389/fimmu.2018.00745 29706964 PMC5908886

[50] Madeddu C Sanna E Gramignano G Tanca L Cherchi MC Mola B. Correlation of leptin, proinflammatory cytokines and oxidative stress with tumor size and disease stage of endometrioid (Type I) endometrial cancer and review of the underlying mechanisms. Cancers (Basel). (2022) 14:e1–e16. doi: 10.3390/cancers14020268 PMC8773675 35053431

[51] Breeur M Ferrari P Dossus L Jenab M Johansson M Rinaldi S. Pan-cancer analysis of pre-diagnostic blood metabolite concentrations in the European Prospective Investigation into Cancer and Nutrition. BMC Med. (2022) 20:351. doi: 10.1186/s12916-022-02553-4 36258205 PMC9580145

[52] Ridgway ND. The role of phosphatidylcholine and choline metabolites to cell proliferation and survival. Crit Rev Biochem Mol Biol. (2013) 48:20–38. doi: 10.3109/10409238.2012.735643 23350810

[53] Knific T Vouk K Smrkolj S Prehn C Adamski J Rizner TL. Models including plasma levels of sphingomyelins and phosphatidylcholines as diagnostic and prognostic biomarkers of endometrial cancer. J Steroid Biochem Mol Biol. (2018) 178:312–21. doi: 10.1016/j.jsbmb.2018.01.012 29360580

[54] Okudaira S Yukiura H Aoki J. Biological roles of lysophosphatidic acid signaling through its production by autotaxin. Biochimie. (2010) 92:698–706. doi: 10.1016/j.biochi.2010.04.015 20417246

[55] Wasniewski T Woclawek-Potocka I Boruszewska D Kowalczyk-Zieba I Sinderewicz E Grycmacher K. The significance of the altered expression of lysophosphatidic acid receptors, autotaxin and phospholipase A2 as the potential biomarkers in type 1 endometrial cancer biology. Oncol Rep. (2015) 34:2760–7. doi: 10.3892/or.2015.4216 26327335

[56] Hishinuma E Shimada M Matsukawa N Saigusa D Li B Kudo K. Wide-targeted metabolome analysis identifies potential biomarkers for prognosis prediction of epithelial ovarian cancer. Toxins (Basel). (2021) 13:e1–e12. doi: 10.3390/toxins13070461 PMC8309959 34209281

[57] Zhang Y Liu Y Li L Wei J Xiong S Zhao Z. High resolution mass spectrometry coupled with multivariate data analysis revealing plasma lipidomic alteration in ovarian cancer in Asian women. Talanta. (2016) 150:88–96. doi: 10.1016/j.talanta.2015.12.021 26838385

[58] Mao X Lei H Yi T Su P Tang S Tong Y. Lipid reprogramming induced by the TFEB-ERRalpha axis enhanced membrane fluidity to promote EC progression. J Exp Clin Cancer Res. (2022) 41:28. doi: 10.1186/s13046-021-02211-2 35045880 PMC8767755

[59] Chen H Wu J Lyu R. Expressions of glycemic parameters, lipid profile, and thyroid hormone in patients with type 2 diabetes mellitus and their correlation. Immun Inflammation Dis. (2024) 12:e1282. doi: 10.1002/iid3.1282 PMC11225078 38967365

[60] Nie Y Chu C Qin Q Shen H Wen L Tang Y. Lipid metabolism and oxidative stress in patients with Alzheimer’s disease and amnestic mild cognitive impairment. Brain Pathol. (2024) 34:e13202. doi: 10.1111/bpa.13202 37619589 PMC10711261

[61] Lorentzen GM Laniewski P Cui H Mahnert ND Mourad J Borst MP. Cervicovaginal metabolome and tumor characteristics for endometrial cancer detection and risk stratification. Clin Cancer Res. (2024) 30:3073–87. doi: 10.1158/1078-0432.ccr-23-2934 PMC11247321 38687603

[62] Moon JY Choi MH Kim J. Metabolic profiling of cholesterol and sex steroid hormones to monitor urological diseases. Endocr Relat Cancer. (2016) 23:R455–67. doi: 10.1530/erc-16-0285 PMC5064754 27580660

[63] Miller WL Bose HS. Early steps in steroidogenesis: intracellular cholesterol trafficking. J Lipid Res. (2011) 52:2111–35. doi: 10.1194/jlr.r016675 PMC3283258 21976778

[64] Orzolek I Sobieraj J Domagala-Kulawik J. Estrogens, cancer and immunity. Cancers (Basel). (2022) 14:e1–e12. doi: 10.3390/cancers14092265 PMC9101338 35565393

[65] Chakraborty S Pramanik J Mahata B. Revisiting steroidogenesis and its role in immune regulation with the advanced tools and technologies. Genes Immun. (2021) 22:125–40. doi: 10.1038/s41435-021-00139-3 PMC8277576 34127827

[66] Brinton LA Trabert B Anderson GL Falk RT Felix AS Fuhrman BJ. Serum estrogens and estrogen metabolites and endometrial cancer risk among postmenopausal women. Cancer Epidemiol Biomarkers Prev. (2016) 25:1081–9. doi: 10.1158/1055-9965.epi-16-0225 PMC4930692 27197275

[67] Chimento A Casaburi I Avena P Trotta F De Luca A Rago V. Cholesterol and its metabolites in tumor growth: therapeutic potential of statins in cancer treatment. Front Endocrinol (Lausanne). (2018) 9:807. doi: 10.3389/fendo.2018.00807 30719023 PMC6348274

[68] Onstad MA Schmandt RE Lu KH. Addressing the role of obesity in endometrial cancer risk, prevention, and treatment. J Clin Oncol. (2016) 34:4225–30. doi: 10.1200/jco.2016.69.4638 PMC5455320 27903150

[69] Blakemore J Naftolin F. Aromatase: contributions to physiology and disease in women and men. Physiol (Bethesda). (2016) 31:258–69. doi: 10.1152/physiol.00054.2015 27252161

[70] Chen P Li B Ou-Yang L. Role of estrogen receptors in health and disease. Front Endocrinol (Lausanne). (2022) 13:839005. doi: 10.3389/fendo.2022.839005 36060947 PMC9433670

[71] Jin X Qiu T Li L Yu R Chen X Li C. Pathophysiology of obesity and its associated diseases. Acta Pharm Sin B. (2023) 13:2403–24. doi: 10.1016/j.apsb.2023.01.012 PMC10326265 37425065

[72] Bruchim I Sarfstein R Werner H. The IGF hormonal network in endometrial cancer: functions, regulation, and targeting approaches. Front Endocrinol (Lausanne). (2014) 5:76. doi: 10.3389/fendo.2014.00076 24904527 PMC4032924

[73] Liu H Zhang L Zhang X Cui Z. PI3K/AKT/mTOR pathway promotes progestin resistance in endometrial cancer cells by inhibition of autophagy. Onco Targets Ther. (2017) 10:2865–71. doi: 10.2147/OTT.S95267 PMC5476755 28652768

[74] Winters SJ Gogineni J Karegar M Scoggins C Wunderlich CA Baumgartner R. Sex hormone-binding globulin gene expression and insulin resistance. J Clin Endocrinol Metab. (2014) 99:E2780–8. doi: 10.1210/jc.2014-2640 25226295

[75] Tabassum R Ruotsalainen S Ottensmann L Gerl MJ Klose C Tukiainen T. Lipidome- and genome-wide study to understand sex differences in circulatory lipids. J Am Heart Assoc. (2022) 11:e027103. doi: 10.1161/jaha.122.027103 36193934 PMC9673737

